# Function and Characteristic Analysis of Candidate PEAR Proteins in *Populus yunnanensis*

**DOI:** 10.3390/ijms241713101

**Published:** 2023-08-23

**Authors:** Ping Li, Jing Wang, Derui Jiang, Anmin Yu, Rui Sun, Aizhong Liu

**Affiliations:** Key Laboratory for Forest Resource Conservation and Utilization in the Southwest Mountains of China (Ministry of Education), College of Forestry, Southwest Forestry University, Kunming 650224, China

**Keywords:** PEAR, Dof, transcription factor, growth regulation, stress resistance, *Populus yunnanensis*

## Abstract

PEAR proteins are a type of plant-specific DNA binding with one finger (Dof) transcription factors that play a key role in the regulation of plant growth, especially during phloem cell growth and seed germination in *Arabidopsis*. However, the identification, characteristics and function of PEAR proteins, particularly in woody plants, need to be further studied. In the present study, 43 candidate PEAR proteins harboring the conserved Zf-Dof domain were obtained in *Populus yunnanensis*. Based on phylogenetic and structural analysis, 10 representative PEAR candidates were selected, belonging to different phylogenetic groups. The functions of PEAR proteins in the stress response, signal transduction, and growth regulation of stem cambium and roots undergoing vigorous cell division in *Arabidopsis* were revealed based on their expression patterns as characterized by qRT-PCR analysis, in accordance with the results of cis-element analysis. In vitro experiments showed that the interaction of transcription factor (E2F) and cyclin indirectly reflects the growth regulation function of PEAR through light signaling and cell-cycle regulation. Therefore, our results provide new insight into the identity of PEAR proteins and their function in stress resistance and vigorous cell division regulation of tissues in *P. yunnanensis*, which may serve as a basis for further investigation of the functions and characteristics of PEAR proteins in other plants.

## 1. Introduction

Transcription factors play important roles in governing plant responses to the environment and regulating growth [1]. The functions of several transcription factors are universal during plant growth and response [2,3,4]. The DNA binding with one finger (Dof) family comprises plant-specific transcription factors, characterized by a single zinc finger structure, which were first found in maize [5,6]. Dof proteins contain a conserved DNA-binding N-terminal and a transcriptional regulation C-terminal, and are found widely distributed in the plant kingdom. The conserved N-terminal contains a Cys2/Cys2 zinc finger DNA binding domain (Dof domain), which can specifically recognize AAAG sequences in the upstream region of target genes [7,8]. 

PEAR proteins are a kind of mobile Dof transcription factor that are enriched with cytokinin in the early protophloem sieve-element cells of *Arabidopsis* root procambial tissue and activate gene expression to promote radial growth [9]. The following PEAR proteins have been characterized in *Arabidopsis*: PEAR1 (*PHLOEM EARLY DOF 1*, known as *DOF2.4*), PEAR2 (PHLOEM EARLY DOF 2, known as DOF5.1), DOF6, TMO6, OBP2, and HCA2 [9]. Before the concept of PEAR proteins was understood, these PEAR members were reported to have important functions in regulating plant growth [9,10,11,12,13,14]. Seed dormancy is an important mechanism of plant self-protection under unfavorable conditions. DNA-BINDING ONE ZINC FINGER 6 (DOF6) acts as a negative regulator of seed germination by forming complexes with downstream proteins (such as MLP329, TCP14, etc.) to regulate primary seed dormancy [10,11]. The DELLA protein RGA-LIKE2 (RGL2) forms a complex with DOF6 that can active *GATA12* (a gene that encodes GATA-type zinc finger transcription factor) to enforce primary seed dormancy [12]. During plant growth, damage and wounding often occur due to biotic and abiotic factors [13]. The Dof transcription factors HIGH CAMBIAL ACTIVITY2 (HCA2), TARGET OF MONOPTEROS6 (TMO6), DOF2.1, and DOF6 are activated at sites of wounding and cell wall damage to promote wound healing and tissue regeneration in *Arabidopsis thaliana* [13]. Glucosinolates play a defensive role against herbivores and microorganisms and are important secondary metabolites in *Capparales* [15]. *AtDof1.1* (OBP2, a DOF transcription factor), is highly expressed in the vasculature of *Arabidopsis*, and is part of a regulatory network in glucosinolate biosynthesis [14]. Cell division and differentiation are important plant responses during wound healing [16]. The periclinal asymmetric cell division of interfascicular parenchyma cells is a basis for the formation of interfascicular cambium, which is important for the transport of water, nutrients, and signaling molecules in higher plants [17]. HCA2 can regulate the formation of cambium and the development of vascular tissues in *Arabidopsis* through affecting periclinal asymmetric cell division [18]. Although the concept of PEAR proteins was only recently proposed, the function of PEAR members have distributed in cell and tissue regulation [9]. 

The study of Dof transcription factors may provide clues for PEAR research. With the development of taxonomic studies, more Dof members with different classifications have been reported, such as five subfamilies found in wheat [19], nine classes in Chinese cabbage [20], four groups in *Populus trichocarpa* [21], four subgroups in pepper [22], nine groups representing four subfamilies in *Jatropha curcas* and *Ricinus communis* [23], four groups in *Medicago truncatula* [24], four classes constituting six clusters in tomato [25], four major clusters in *Oryza sativa* and *Arabidopsis thaliana* [26], and eight groups representing four subfamilies in *Manihot esculenta* [27]. Their classification may give some clues for functional research. Dof is also distributed in various kinds of growth regulation processes in plants. For example, SIDOF10 regulates vascular tissue formation in the process of fruit setting, especially in young tissue undergoing vigorous cell division [28]. OBP1, a Dof transcription factor in *A. thaliana*, is a transcriptional regulator of key cell cycle genes and is involved in the control of cell division via developmental signaling [29]. SCAP1, a Dof transcription factor, regulates the expression of genes involved in stomatal functions and morphogenesis, which in turn regulate the essential processes of stomatal guard cell maturation [30].

PEAR is a kind of Dof transcription factor that contains characteristic Zf-Dof domains. In woody plants, Dof transcription factors have been identified in some species [21,31]. But, the research on PEAR was lacking. Research is needed on the function and classification of PEAR, especially in woody plants, to explain their potential role in growth regulation in perennial plants. *Populus yunnanensis*, which is an important economic tree, is also important for ecological restoration, especially in the mining area of Southwest China [32,33]. To identify and explore the functions of PEAR proteins in woody plants, we performed a homology comparison and characteristic analysis of *Arabidopsis* PEAR proteins and found 43 candidate PEAR proteins in *P. yunnanensis*. Then, we screened for PEAR proteins using enrichment data of *P. trichocarpa* (a poplar that has a close evolutionary relationship with *P. yunnanensis*), and intraspecific and interspecies collinearity. After the expression analysis and verification of interactions, we confirmed the functions of PEAR proteins in cell growth regulation and ABA response in *P.yunnanensis*.

## 2. Results

### 2.1. Genome-Wide Identification of PEAR Candidates

After a sequence similarity search of *P. yunnanensis* with 6 *Arabidopsis* PEAR proteins, 43 candidate PEAR proteins were obtained (Table 1 and Table S1). All 43 *P. yunnanensis* candidate PEAR proteins contained conserved Zf-Dof domains [5]. The length and molecular weight of *P. yunnanensis* PEAR proteins varied, but the Zf-Dof domains of these candidates were conserved and comprised 57–58 amino acids, except for Poyun21040, with 44 amino acids. Of the 43 candidate PEAR proteins, 14 candidates were weakly acidic and the other 29 candidates were neutral or basic. The subcellular location prediction of PEAR candidates revealed that the majority of PEAR candidates were localized in the nucleus, except for Poyun30052, Poyun30013 (mitochondria), and Poyun14051 (cytoplasm).

### 2.2. Sequence Characteristics and Phylogenetic Relationship of PEAR Candidates

**Figure 1:**
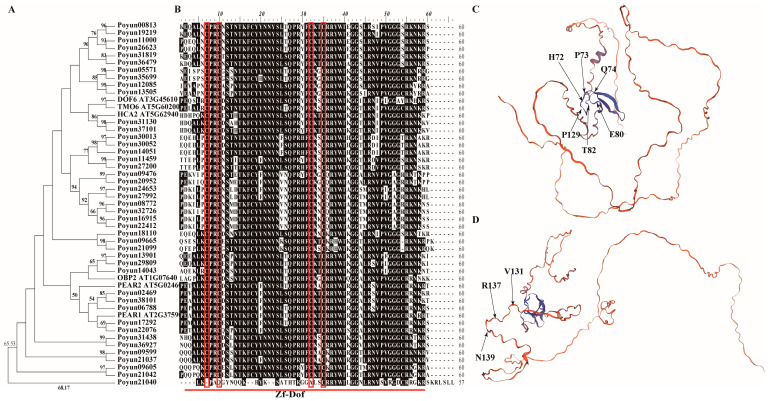
Sequence alignment and structure analysis of AtPEAR proteins and their candidates in *P. yunnanensis*. (**A**) Maximum likelihood phylogenetic tree of 6 AtPEAR and 43 PEAR candidate proteins. Amino acid sequences were aligned with Clustal W, and phylogenetic tree was constructed using MEGA 11. (**B**) Multiple sequence alignment of AtPEAR proteins and their candidates in *P. yunnanensis* with BioEdit. Red underline and letters denote domains of PEAR proteins. Four representative cysteine residues of zinc-finger structure are indicated with red box. Black shading indicates >90% in corresponding position. (**C**,**D**) Protein structure of Zf-Dof domain in representative PEAR candidate proteins in *P. yunnanensis*. Secondary structure of Poyun02469 (PEAR2 homolog) (**C**) and Poyun31130 (HCA homolog) (**D**) predicted using Expasy-ProtParam. The Poyun02469 model was built using a AOA4V6AAQ1.1.A template (*Populus alba*) (84.47% sequence identity, 1.00 coverage). The Poyun31130 model was built using a I1KCF8.1.A template (*Glycine max*) (80.26% sequence identity, 0.99 coverage). The arrows labeled amino acids in the model were conserved in the domain helix (His72, Pro73, Gln74, Glu80, Thr82 and Pro129, (**C**)) and domain corner (Val131, Arg137, and Asn139, (**D**)).

To obtain the sequence characteristics of PEAR candidates in *P. yunnanensis*, all 43 proteins and 6 AtPEAR protein sequences were aligned and analyzed using BioEdit [35]. All proteins contained a conserved Zf-Dof domain (Table 1). Based on their phylogenetic relationships, the sequences appeared to be diverse (Figure 1A,B). Except for DOF6 and TMO6, homologous PEAR candidates were found for the other four AtPEAR proteins. Poyun31130 and Poyun37101 shared a common evolutionary branch with AtHCA2. Poyun31130 and Poyun02469 were conserved in all domain corners according to models built using Dof zinc finger protein templates (from *Glycine max* and *Populus alba*) (Figure 1C,D). The amino acids of His72, Pro73, Gln74, Glu80, Thr82, and Pro129 in the domain helix of AtHCA2 in *P. yunnanensis* were found to have been replaced by amino acids with the same isoelectric point values. Poyun13901, Poyun29809, and Poyun14043 shared the same evolutionary branch as AtOBP2. The sequence alignment of AtOBP2 and its candidates revealed sequence diversity; even some domain-corner amino acids showed variation, such as Val131, Arg137, and Asn139, which were replaced by amino acids with the same physical and chemical properties. Poyun02469, Poyun38101, Poyun06788, Poyun17292, and Poyun22076 showed similarity with AtPEAR1 and AtPEAR2. The sequence similarity in the domain of AtPEAR1 and AtPEAR2 branch proteins exceeded 90%.

### 2.3. Phylogenetic Relationships and Molecular Characterization of PEAR Candidates in P. yunnanensis

**Figure 2:**
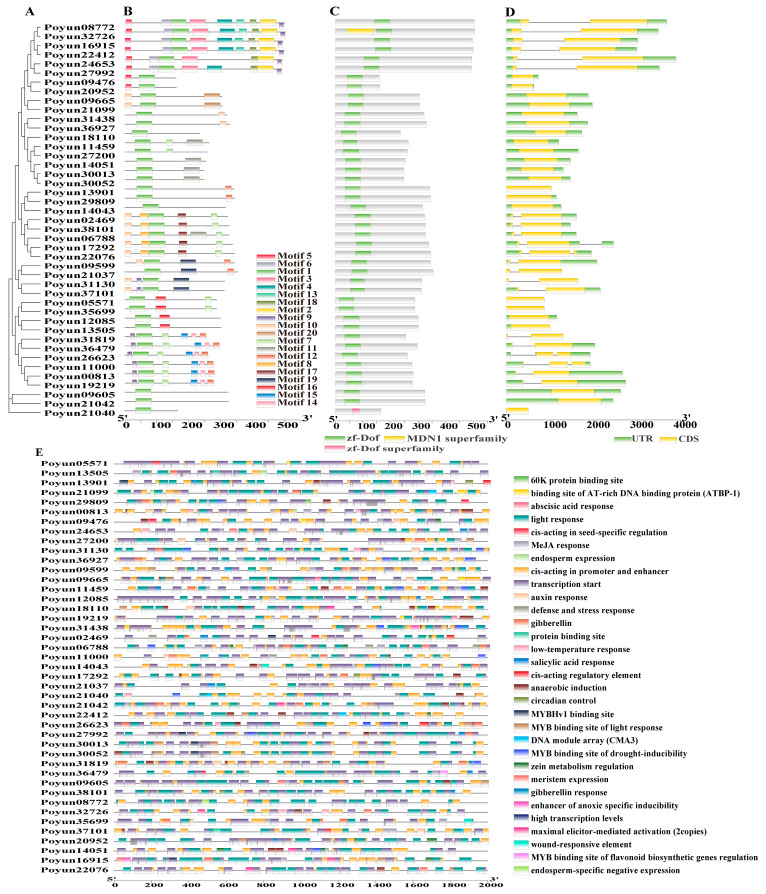
Phylogenetic tree, motifs, cis-elements, and structure of PEAR candidates in *P. yunnanensis*. (**A**) Phylogenetic tree constructed based on maximum likelihood method. (**B**) Conserved motifs of PEAR candidates as predicted using Multiple Em for Motif Elicitation (MEME). A total of 15 conserved motifs are represented by colored boxes. (**C**) Schematic diagram of Zf-Dof domains in PEAR candidates; green boxes indicate Zf-Dof domains. (**D**) Gene structure of PEAR candidates. Yellow boxes indicate CDS, and green boxes indicate non-coding sequences. (**E**) Cis-elements of PEAR candidates within 2000 bp upstream sequences.

The multiple sequence alignment of 43 PEAR candidates in *P. yunnanensis* was performed to construct a maximum likelihood phylogenetic tree. The tree revealed that the 43 PEAR candidates were classified into four main branches (Figure 2A). As shown in Figure 2, motif1, which is characteristic of Zf-Dof domain, was conserved and identified in all PEAR candidates (Figure 2B, Table S2). The AtHCA2 candidates (Poyun31130 and Poyun37101) contained conserved motifs 9, 10, and 19, but not motif 1. The AtOBP2 candidates presented different motif distributions. Poyun29809 and Poyun14043 contained conserved motif 12, and motif 1 was not present in Poyun13901. The AtHCA2 candidates (Poyun31130 and Poyun37101) contained conserved motifs 9, 10, and 19 but not motif 1. The PEAR candidates contained conserved motifs 1, 7, 8, 10, and 17, and Poyun06788 also contained motif 11. Protein structure analysis of the AtHCA2 candidates revealed one conserved Zf-Dof domain, except Poyun21040, which contained a subfamily domain. The length of the proteins and the location of the domains were found to vary according to the phylogenetic tree branch (Figure 2C, Table 1). The length of AtPEAR1, AtPEAR2, AtOBP2 and AtHCA2 was in the middle of the range for all candidate proteins. The AtPEAR1 and AtPEAR2 candidates had longer N-terminal (more than 95 amino acids) than other candidates. The AtOBP2 candidates had a shorter N-terminal, which was less than 35 amino acids. The AtHCA2 candidates had an N-terminal of 75 amino acids. To reveal the coding characteristic of PEAR protein candidates, their gene structure was investigated by comparing their DNA sequences. The results showed that the gene structures varied according to the phylogenetic branch (Figure 2D). Only AtOBP2 candidates harbored one long intron, which separated the exon into two parts. The AtPEAR1 candidates harbored two introns and three exons. The AtPEAR2 candidates harbored a short intron.

### 2.4. Cis-Elements in Promoter Region of Candidate Proteins of AtPEAR Coding Genes

To investigate the regulatory mechanism, we searched for possible cis-elements in the promoter region of the candidate proteins of AtPEAR coding genes (Figure 2E, Table S3). Many of the cis-elements involved in hormone signaling, transcription regulation, and stress response were predicted using PLACE. The most frequent cis-elements found were those involved in light response, which suggests the important function of light signals. Cis-elements related to transcription initiation, promoters, and enhancers revealed the strong transcriptional regulation capability of PEAR candidates. ABRE is another widespread cis-element involved in abscisic acid (ABA) response, which was indicative of the function of PEAR candidates in stress response and growth regulation [36]. The presence of ARE, TGA-elements, TC-rich repeats, P-box, and TGACG-motifs, which are characteristic of cis-elements involved in hormone signaling, stress response and repair, reveals the function of PEAR candidates in anaerobic induction, auxin response, defense and stress response, gibberellin, MeJA response, and salicylic acid response [37,38,39]. Other cis-elements involved in growth regulation, such as CAT-box, O2-site, MBS, G-box, LTR, circadian clock, and AT-rich elements, have also been detected, which are involved in the functions of meristem expression, zein metabolism regulation, MYB binding site of drought inducibility, MYB binding site of flavonoid light regulation, maximal elicitor-mediated activation, low-temperature response, and circadian rhythm [40,41]. On the other hand, cis-elements involved in light response, transcription regulation, stress response, growth regulation, hormone signaling, and the binding site of AT-rich DNA binding protein (ATBP-1) were found to be enriched in the candidates AtOBP2, AtHCA2, and AtPEAR1.

### 2.5. Expression Patterns of PEAR Candidates in Different Tissues and Stress Treatment

**Figure 3:**
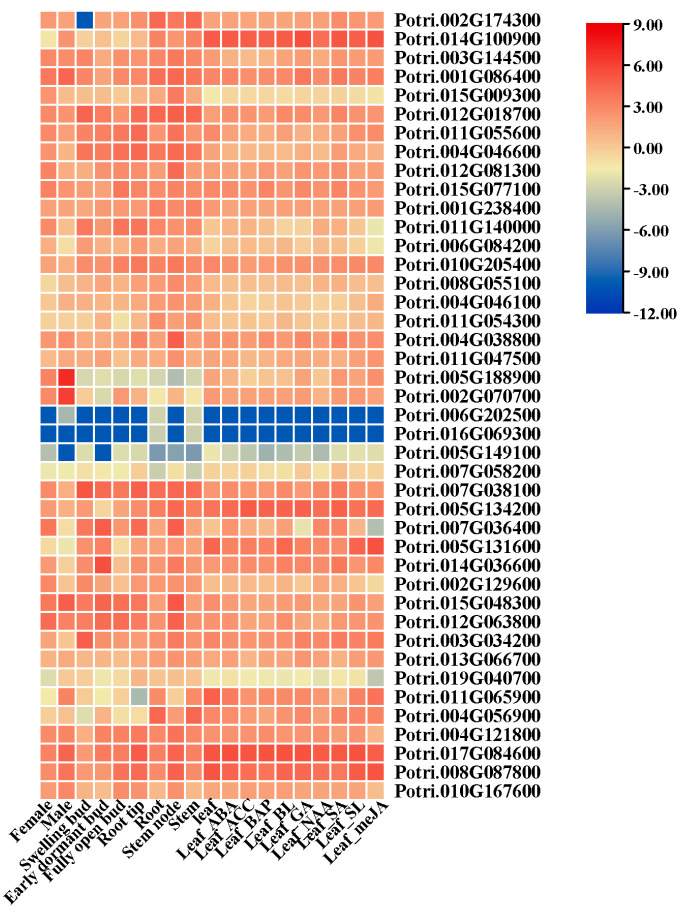
Heatmap of expression profiles of *P. yunnannesis* candidate PEAR homolog proteins in *P. trichocarpa*. Expression data based on log2 (FPKM) values at different development stages (female and male plant leaves, swelling, early dormant and fully open buds), organs (bud, leaf, root, root-tip, stem), and phytohormone treatments (ABA, ACC, BAP, BL, GA, NAA, SA, SL, and meJA) from Phytozome 13.

To investigate the function of PEAR candidates in poplar, we obtained publicly available expression data for 42 homologs of *P. yunnanensis* PEAR candidates in *P. trichocarpa*, which has the closest evolutionary relationship to *P. yunnanensis* (Figure S1). As shown in Figure 3, the expression of PEAR candidates varied in different tissues and treatments (Table S4). *Poyun26623* (*Potri.014G100900*), *Poyun32726* (*Potri.017G084600*), and *Poyun22412* (*Potri.008G087800*) were significantly enriched under treatment with different kinds of hormones, and were linked with stress response cis-elements. *Poyun36927* (*Potri.012G063800*), *Poyun31438* (*Potri.015G048300*), *Poyun30052* (*Potri.007G036400*), *Poyun09599* (*Potri.004G046600*), *Poyun21037* (*Potri.011G055600*), *Poyun36479* (*Potri.012G018700*), and *Poyun00813* (*Potri.001G086400*) were enriched in fast-growing tissues such as root tip and bud. The AtPEAR1 and AtPEAR2 candidates *Poyun02469* (*Potri.001G238400*), *Poyun38101* (*Potri.011G140000*), *Poyun06788* (*Potri.006G084200*), *Poyun17292* (*Potri.010G205400*), and *Poyun22076* (*Potri.008G055100*) were enriched in young and fast-growing tissues (bud, stem, and root-tip). The AtHCA2 candidates were also characteristically enriched in fast-growing tissues, such as bud and stem node (*Poyun37101* (*Potri.012G081300*)) and during phytohormone treatment (*Poyun31130* (*Potri.015G077100*)). The AtOBP2 candidates *Poyun29809* (*Potri.007G058200*) and *Poyun13901*(*Potri.005G149100*) maintained low expression levels in all test samples; however, Poyun14043 was significantly enriched in fast-growing tissues such as bud, root tip, and stem. The commonality here is that all AtPEAR candidates were significantly enriched in fast-growing tissues.

**Figure 4:**
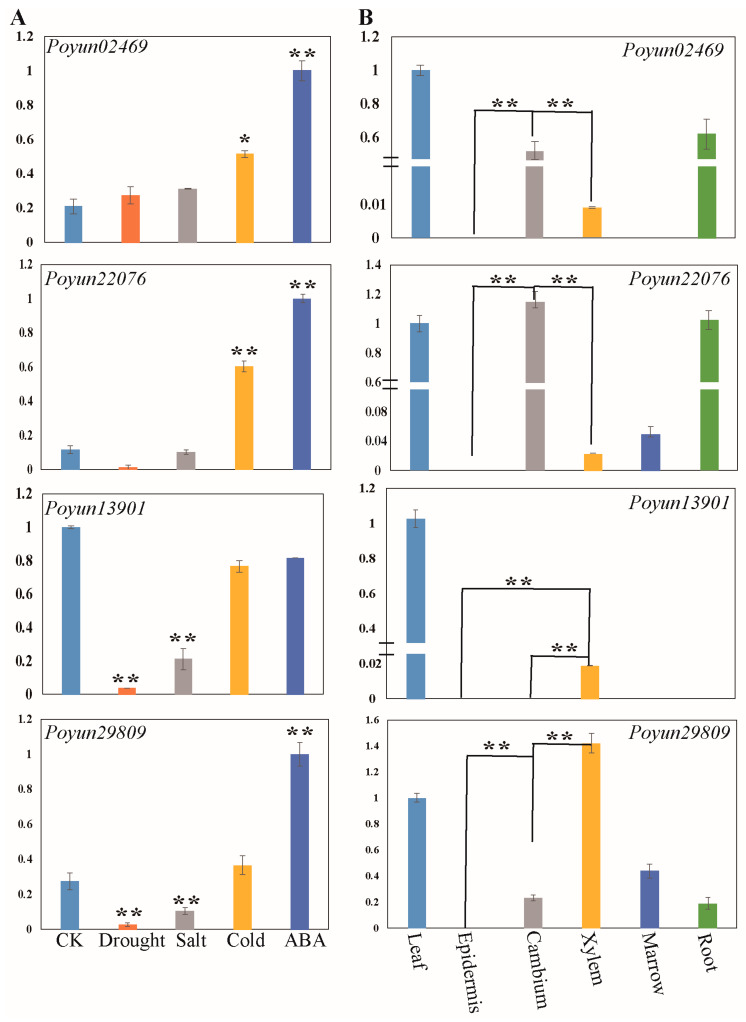
Expression patterns of four representative candidate PEAR genes by qRT-PCR. (**A**) Under stress treatment. CK: untreated control leaves; drought: two days no-watering treatment; salt: 150 mM NaCl (dissolved in 100 mL water) were added to cultured plant pots for 1 day; cold: seedlings grown in pots were cultured at 4 °C for 1 day; ABA: 50 μmol/mL abscisic acid (ABA) (dissolved in 50 mL water) were sprayed on plant material for 1 day. (**B**) Different tissues collected from leaves, epidermis cambium, xylem, and marrow were collected from two months old cutting seedlings. The 2^−ΔΔCT^ method was used for calculated relative expression changes of selected *PEAR* genes, with respect to reference gene (EF1) [42]. All qRT-PCR analyses were conducted with three replicates. Data represent means of three biological replicates and error bars indicate ± SE (*n* = 3). Asterisks indicate significant differences by Tukey’s LSD test (* *p* < 0.05, ** *p* < 0.01). Significant relative to control in (**A**); significant contrast samples are connected by lines between tissues in (**B**).

To verify whether candidate PEAR proteins would respond to stress treatment and were involved in growth regulation, we simulated stress conditions and isolated different tissues of *P. yunnannesis*. The relative expression of the representative candidate PEAR genes during stress response and tissue differentiation was further analyzed using qRT-PCR analysis (Figure 4). The PEAR candidates *Poyun02469* and *Poyun22076* were upregulated under stress treatment, especially under cold and ABA treatment (Figure 4A). On the other hand, *Poyun02469* and *Poyun22076* were significantly highly induced in the stem cambium, in contrast to the rest of the stem tissues and young roots (Figure 4B). The AtOBP2 candidates *Poyun13901* and *Poyun29809* responded negatively to drought and salt stress (Figure 4A). On the other hand, *Poyun13901* and *Poyun29809* were highly induced in the xylem, in the contrast to the stem tissues (Figure 4B).

### 2.6. Chromosomal Localization and Collinearity Analysis of PEAR Candidates

The chromosome distribution analysis of the *P. yunnanensis* genome revealed that the 43 PEAR candidates were unequally distributed among 18 chromosomes, except for chromosome (Chr) 16 (Table S5). Chr 3 and 5 contained the maximum number of PEAR candidates (four). The AtHCA2 candidate *Poyun37101* was found to be located on Chr 18, and *Poyun31130* was located on Chr 14. AtOBP2 candidates *Poyun13901* and *Poyun14043* were located on Chr 5, and *Poyun29809* was located on Chr13. The AtPEAR candidates were distributed on Chr 1 (*Poyun02469*), Chr 2 (*Poyun06788*), Chr 6 (*Poyun17292*), Chr 9 (*Poyun22076*), and Chr 19 (*Poyun38101*).

**Figure 5:**
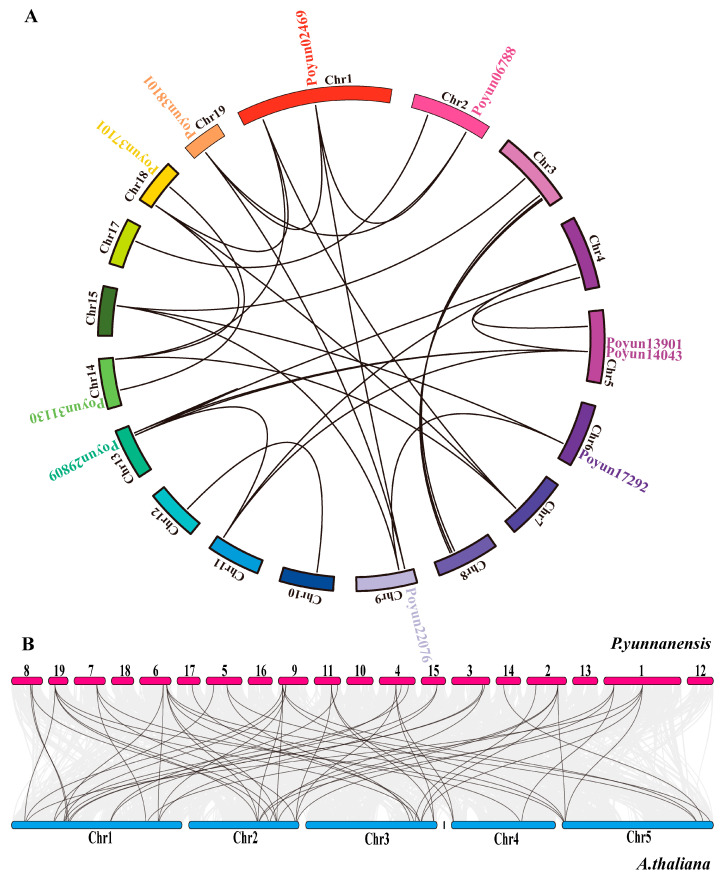
Gene duplication events and collinearity of candidate PEAR genes of (**A**) *P. yunnanensis* and (**B**) between *P. yunnanensis* and *A.thaliana*. Collinearity pairs of candidate PEAR genes are connected with black lines. Chromosome numbers are indicated near each chromosome boxes. The collinearity pairs were list in Table S5.

Gene duplication events are always associated with plant evolution, which is one of the main phenomena underlying the expansion of gene families [43]. Synteny analysis revealed that 36 PEAR candidates in *P.yunnanensis* demonstrated genomic synteny (Figure 5, Table S5). A total of 40 PEAR candidate pairs arose from genome duplication events. In the collinearity region of AtPEAR1 candidates (*Poyun17292* and *Poyun22076*) and AtPEAR2 candidates (*Poyun02469*, *Poyun38101*, and *Poyun06788*), the repeated collinearity relationships found among them may have arisen from genome duplication events. There were 56 gene pairs between *P.yunnanensis* PEAR candidates and the *A.thaliana* genome. All AtPEAR candidates had more than one collinear gene in the *A.thaliana* genome, which indicates that there was conservation of PEAR between species and following genome duplication events of *PEAR* genes in *P. yunnanensis*. *Poyun31130*, an AtHCA2 candidate, exhibited collinearity with AtHCA2 (*AT5G62940*), providing evidence of the conservation of HCA2 across species.

### 2.7. Interaction Networks of PEAR Candidates

**Figure 6:**
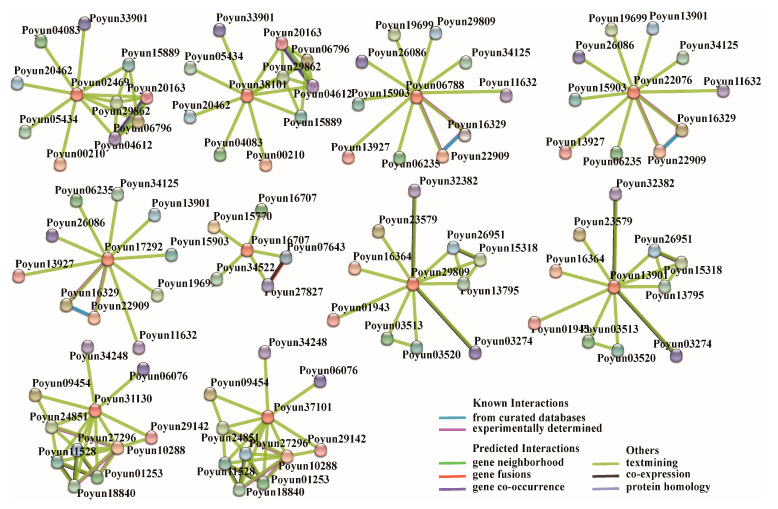
Predicted network of protein–protein interactions of PEAR candidates of *P. yunnanensis* using STRING. Spheres represent interaction proteins. The orange proteins in the center represented ten *P. yunnanensis* PEAR proteins. Color lines represent different types of interaction relationships between proteins. Known interactions: interaction relationships verified by curated databases (blue) or experiments (purple); predicted interactions: interaction relationships predicted by gene neighborhood location (green), gene fusion (red), or gene co-occurrence (blue violet); others: possible interactions of proteins by text mining (olive), co-expression (black), or protein homology (gray-purple).

**Figure 7:**
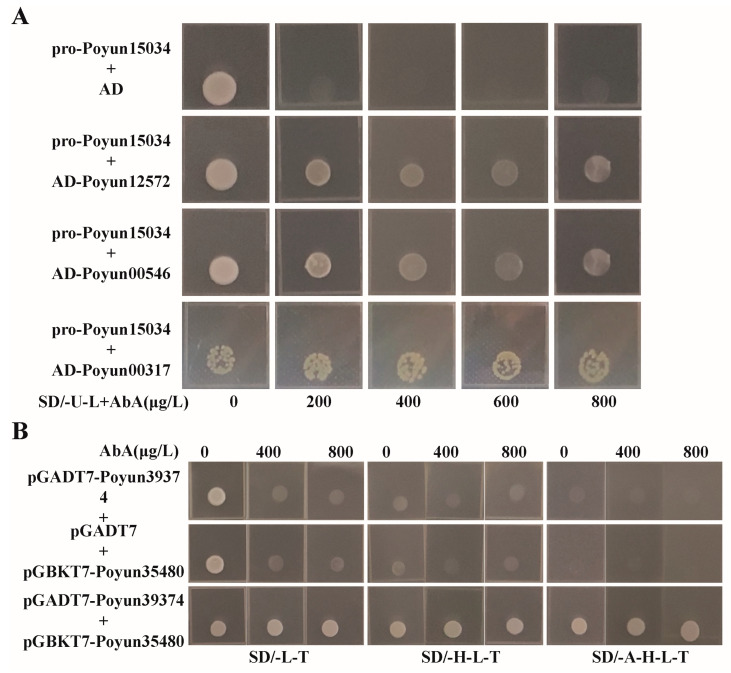
Protein–gene and protein–protein interactions detected using Y1H and Y2H assays. (**A**) In Y1H assay, selected upstream promoter sequence of *Poyun15034* (cyclin) was ligated to pAbAi (pro-Poyun15034), and coding sequences of *Poyun12572*, *Poyun00546,* and *Poyun00317* genes (E2F) were ligated to pGADT7 (AD). pro-Poyun15034 + AD was used as negative control of Y1H. (**B**) In Y2H assay, coding sequence of *Poyun39374* (E2F) was ligated to activation domain (AD, as pGADT7-*Poyun39374*), and coding sequence of *Poyun35480* (E2F) was fused to GAL4 DNA-binding domain, (BD, as pGBKT7-*Poyun35480*). pGADT7-*Poyun39374*+ pGBKT7 and pGADT7 + pGBKT7-*Poyun35480* were used as negative control of Y2H. SD/-U-L, SD/-L-T, SD/-H-L-T, and SD/-A-H-L-T represent SD-Sec-Leu, SD-Leu-Trp, SD-His-Leu-Trp, and SD-Ade-His-Leu-Trp medium, respectively. Different concentrations of Aureobasidin A (AbA, µg/L) were added for screening.

PEAR candidates are a type of Dof transcription factor that can form homodimers with other genes and proteins using their recognized DNA binding domain [13]. The predicted protein–protein interaction network based on representative *Populus* PEAR candidates is shown in Figure 6. It was observed that most PEAR candidates interact with other transcription factors (Table S6), such as homeobox-leucine zipper and basic helix-loop-helix transcription factors. Aside from transcription factors, pectate lyase (which is related to the cell wall), DNAJ heat shock family proteins, flavin-binding kelch domain proteins, and kinesin-like proteins, were also predicted to interact with PEAR candidates. From the interaction results of candidate PEAR proteins, we could speculate that cell division and repair are important for their functioning. To further verify the protein–protein interactions of cell division proteins and PEAR candidates, we performed Y1H and Y2H experiments. The representative genes were cloned into experiment vectors. Unfortunately, we did not observe any direct interaction of these cell division proteins with PEAR candidates. During the experiment assessing protein interactions, we found that cyclin protein (Poyun15034) can directly interact with E2F transcription factors (Poyun05546 and Poyun12575) at the protein and gene level, possibly forming complexes with PEAR proteins to influence cell division (Figure 7).

## 3. Discussion

Plant-specific Dof transcription factors are important for stress response, hormone signal regulation, and plant growth regulation [7,28,29,30,44,45]. As Dof transcription factors, PEAR proteins were found to be functional in early protophloem cells and cytokinin signaling [9]. The typical characteristic of Dof is a single zinc finger structure containing a DNA-binding N-terminal and a transcriptional regulation C-terminal [6]. Sequence similarity was the basis of protein research across plant species [26]. A total of 43 candidate PEAR proteins in *P. yunnanensis* were obtained using BLAST (E-value of 1× 10^-5^) with *Arabidopsis* PEAR proteins (Table S1) [9,26]. In contrast to *Arabidopsis* (36 AtDofs) and rice (30 OsDofs), *P. yunnanensis* has more candidate PEAR proteins (Dof transcription factors) [26]. Wheat (96 TaDofs) and Chinese cabbage (76 BraDofs) contained more Dof transcription factors than *P. yunnanensis* [19,20]. As the same genus species, *P. trichocarpa* (41 PtrDofs) and *P. yunnanensis* (43 Dofs) contained different numbers of Dofs, which revealed the Dof transcription factors were various according to different species [21]. The different amino acids lengths and physicochemical characteristics of the 43 candidate PEAR proteins revealed the differentiation (Table 1). To analyze the relationship between PEAR proteins and Dof transcription factors, we built a phylogenetic tree using protein sequences of candidate PEAR proteins in *P. yunnanensis* and *Arabidopsis* PEAR proteins (Figure 1A) [9]. The physiological tree shows the evolutionary relationship and similarity between AtPEAR and *P. yunnanensis* PEAR candidates [46]. The Zf-Dof domain common to 42 *P. yunnanensis* candidate PEAR proteins (except for poyun21040) verified the authenticity of the PEAR proteins (or Dof transcription factors) (Figure 1B) [5,6].

The homologs of six AtPEAR proteins were distributed in different groups with *P.yunnanensis* PEAR candidate proteins. Compared to AtHCA2, no homologs of AtDOF6 and AtTMO6 were found in *P. yunnanensis*. In *Arabidopsis*, AtDOF6 was regulator of primary seed dormancy [10]. AtTMO6 was involved in cell wound healing and tissue regeneration after squeezing and cutting treatment [13]. The missing homologs of AtDOF6 and AtTMO6 revealed their functional species was specific or focused on herbs with seed dormancy [10,11,13]. AtHCA2 was also involved in tissue development through interfascicular cambium formation and vascular tissue development [18]. The missing homologs of AtTMO6 may reveal functional complementation of AtTMO6 and AtHCA2 in *P. yunnannesis* according to their functional similarity in *Arabidopsis* and evolutionary similarity in *P. yunnanensis* (Figure 1A) [13]. The sequence similarity was consistent with the phylogenetic tree (Figure 1B). Poyun02469, Poyun38101, Poyun06788, Poyun17292, and Poyun22076 share the same phylogenetic branch as AtPEAR1 and AtPEAR2, which share more sequence similarity compared to the other PEAR candidates (Figure 1A,B,D). Poyun31130 and Poyun37101 were found to be similar to AtHCA2 during their Zf-Dof domain structure, phylogenetic relation, protein characteristics, and substitution of amino acids (Table 1, Figure 1B,D). Poyun13901, Poyun29809, and Poyun14043 had phylogenetic similarity with AtOBP2, Poyun13901, and Poyun29809 exhibited more similar domain characteristics, phylogenetic relationships, and sequence similarity. All three AtOBP2 homolog PEAR candidates are basic proteins that contain identical amino acid substitutes. The sequence and structure similarity may indicate the functional similarity [47] and redundancy [48] between homologs. The homologs of AtHCA2 and AtPEAR contained similar protein building models (Dof) from different plant species (*Glycine max* and *Populus alba*), which indicated the variability between candidate PEAR proteins (Figure 1C,D) [49]. On the other hand, the different similarity of *P. yunnanensis* PEAR homologs with *Arabidopsis* revealed the functional differentiation of Herbs and woody plants [21,50].

To verify the structure of and relationship between 10 representative PEAR candidates and other candidate proteins, a phylogenetic tree was constructed and characteristic analysis was performed with 43 candidate REAR protein (Dof transcription factor) sequences (Figure 2). Consistent with the 42 Dof transcription factors of *P. trichocarpa*, 43 candidate PEAR proteins with conserved Zn-Dof domains clustered into four groups based on their phylogenetic relationships and gene and protein structure (Figure 2) [21]. *Capsicum annuum* L. [22], *R. communis* [23], *M. truncatula* [24], *O. sativa* [26], *A. thaliana* [26], and *M. esculenta* [27] also contained four groups of Dof transcription factors according to phylogenetic analysis, which showed the conserved classification. The phylogenetic relationship of PEAR candidates indicated that the AtOBP2 and AtPEAR homologs shared the same group and branch, but the conserved motifs and gene structure of the homologs varied, which implied the functional variability (Figure 2, Table S2) [49]. The homologs of AtHCA2 were also found to differ from the homologs of AtOBP2 and AtPEAR in terms of phylogenetic relationship, conserved motifs, and gene structure (Figure 2). Their being on the same evolutionary branches verified the evolutionary similarity of the proteins; however, the evolutionary differentiation was predicted with different structure subunits [27].

Many cis-elements were predicted to be involved in plant growth and development, signal transduction, and stress response [51]. The highly enriched cis-elements about light response, transcription enhancement, and ABRE revealed the function of PEAR candidates in plant growth regulation and stress response (Figure 2D, Table S3) [4,36,37,38,39,40]. To further speculate on the function of PEAR candidates, we collected the expression patterns of all PEAR candidate homologs of *P. yunnanensis* in *P. trichocarpa* (Figure 3, Table S4). Homologs of AtHCA2, AtOBP2, AtPEAR1, and AtPEAR2 were found to be highly enriched in fast-growing tissues, especially in root tips and buds, which was consistent with their functions of tissue growth regulation in *Arabidopsis* (Figure 3, Table S4) [9,14,18]. The high expression of homologs of AtPEAR and AtOBP2 in *P. yunnanensis* also verified their function on young tissue with rapidly separating cells (stem cambium and xylem), which correlates with their function of cell differentiation in *Arabidopsis* (Figure 4) [9,14]. The unequal chromosome distribution of 43 PEAR candidates revealed their different and duplication functions (Table S5) [20]. Collinearity analysis of the inter-chromosomal relationships of *P. yunnanensis* PEAR homologs revealed the gene duplication events of the PEAR candidate genes of *P. yunnannesis*, which were correlated with functional similarity (Figure 5A) [46]. The collinearity relationship between different homologs of AtHCA2, AtPEAR1, and AtPEAR2 across species (*Arabidopsis* and *P. yunnannesis*) also revealed the duplication of different copies of genes with the same function and expression pattern, which may be correlated with functional redundancy (Figure 5B) [43].

The interaction of transcription factors and target genes or proteins is an important regulation mechanism of plant growth [52]. Most of the *P. yunnanensis* candidate PEAR proteins were predicted to interact with other transcription factors and proteins (Table S6). Specifically, for the homologs of AtPEAR1, AtPEAR2, AtOBP2 and AtHCA2, common interaction proteins are bHLH transcription factor, DNAJ domain protein, flavin-binding kelch domain protein and kinesin-like protein (Figure 6). The bHLH transcription factors represented one of the largest families of transcription factors and are involved in biosynthesis, metabolism, transduction of plant hormones, and especially pleiotropic regulation in growth regulation and stress response [53]. The interaction between *P. yunnanensis* PEAR candidates and bHLH transcription factors may be involved in regulation on young tissues and stress response (Figure 4 and Figure 6). The function of DnaJ homolog genes (*KAM2*/*GRV2*) in the determination of embryonic growth axis in *Arabidopsis* was in accord with the high expression of PEAR candidates in young tissues with vigorous cell division (Figure 4 and Figure 6) [54]. FLAVIN-BINDING, KELCH REPEAT, F-BOX protein (FKF1) protein is essential for light signaling in *Arabidopsis*, and provides new information on growth regulation function of PEAR candidates in *P. yunnanensis* [55]. The predicted interaction of the kinesin-like protein occurred during cell synchronization and cell-cycle regulation, which also provides evidence for the function on cell growth and differentiation regulation of *P. yunnanensis* PEAR candidates [56]. Unfortunately, we did not observe a direct interaction between PEAR candidates and predicted proteins in the in vitro validation testing. However, the interaction between cyclin and E2F during the interaction testing of PEAR candidates revealed that the regulation and progression of the cell cycle are important for the function in growth regulation of *P. yunnanensis* PEAR candidates (Figure 7) [57,58]. Our study found ten *P. yunnanensis* PEAR candidate proteins using phylogenetic and characteristic analysis. The expression and interaction results demonstrate that *P. yunnanensis* PEAR candidate proteins play a crucial role in stress response and growth regulation, especially in young tissues with vigorous cell division. This paper reveals the PEAR proteins in *P. yunnanensis* for further study.

## 4. Materials and Methods

### 4.1. Identification of Candidate PEAR Proteins

To identify candidate PEAR proteins in *P. yunnanensis*, a sequence similarity search was performed with *Arabidopsis* PEAR (AtPEAR) proteins using Basic Local Alignment Search Tool (BLAST) version ncbi-blast-2.7.1 (available at https://blast.ncbi.nlm.nih.gov/Blast.cgi; accessed on 23 March 2023) [9]. The *P. yunnanensis* databases in BLAST were from the newly released sequencing data of NCBI (accession number PRJCA008692) [34]. A total of 43 candidate PEAR proteins were obtained in *P. yunnanensis* based on their similarity to those in *Arabidopsis* (with E-value of 1e-5, calculated with sequence similarity by BLAST) [26]. The protein sequences of the identified PEAR candidates in *P. yunnanensis* were subjected to domain analysis using PFAM (https://pfam.xfam.org/; accessed on 21 April 2023) and the Batch CD-Search Tool (https://www.ncbi.nlm.nih.gov/Structure/bwrpsb/bwrpsb.cgi; accessed on 21 April 2023). The features of protein domains were analyzed using Expasy-ProtParam (https://web.expasy.org/protparam/; accessed on 7 April 2023). The subcellular localization of the candidate PEAR genes was predicted using WOLF PSORT (https://wolfpsort.hgc.jp/; accessed on 7 April 2023).

### 4.2. Phylogenetic Analysis of PEAR Candidates in P. yunnanensis

The phylogenetic tree of AtPEAR proteins and candidate PEAR proteins of *P. yunnanensis* was constructed in MEGA11 (http://www.megasoftware.net/history.php; accessed on 21 April 2023) using the maximum likelihood (ML) method [59,60]. The bootstrap value of each branch reflects the percentage of 1000 replicate trees containing that branch. The rooted *Populus* tree was obtained using OrthoFinder method with a phylogenetic inference of orthologs isolated from Populus species gene sequences, which were downloaded from NCBI (https://www.ncbi.nlm.nih.gov/; accessed on 23 June 2022) [61]. Sequence alignments results were obtained using Bioedit [62] with full candidate PEAR protein sequences, after multiple sequence alignment (1000 bootstraps) using ClustalW [63].

### 4.3. Gene Structure, Conserved Motifs, Domains and Cis-Elements of Candidate PEAR Genes

The gene structure of 43 candidate PEAR genes of *P. yunnanensis* was analyzed using the Gene Structure View package of TBtools (v1.098691) with gff3 data [34]. The conserved motifs of PEAR candidates were detected using Multiple Em for Motif Elicitation (MEME) (https://meme-suite.org/meme/tools/meme; accessed on 31 March 2023). The conserved cis-elements in the promoters of candidate PEAR genes, corresponding to the 2 kb sequence upstream before the translational start codon, were detected using the PLACE database (https://www.dna.arc.go.jp/PLACE/; accessed on 31 March 2023).

### 4.4. Expression Pattern of PEAR Candidates in P. trichocarpa

*Populus trichocarpa* transcriptome data, obtained from Phytozome 13 (https://phytozome-next.jgi.doe.gov/; accessed on 5 May 2023), were used to analyze the expression profile of PEAR candidates in *P. trichocarpa* at different development stages (female and male plant leaves, swelling, early-dormant and fully open buds), organs (bud, leaf, root, root-tip, stem) and phytohormone treatments (ABA, ACC, BAP, BL, GA, NAA, SA, SL, and meJA). The expression values in the heatmap were calculated using the log2 of the Fragments Per Kilobase of transcript per Million mapped reads (FPKM) values from Phytozome 13. The heatmap of candidate PEAR genes of *P. trichocarpa* was obtained using HeatMap package of TBtool [64].

### 4.5. Plant Materials and qRT-PCR Assays

The cutting plant materials were collected from *P. yunnanensis* growing for one years at Kunming (E102.74°, N25.17°). Cuttings (about 25 cm) were cultured for two months in a green house (16 h light at 25 °C/8 h dark at 18 °C) of Southwest Forestry University with mixed nutrient medium pots (humus: quartz sand: perlite at 3:1:1) [65]. For the different tissue tests, fresh healthy leaves, stems (including detached epidermis, cambium, xylem, and marrow), and roots were collected from two-month-old cutting seedlings. At least three biological replicates of plant tissues were immediately frozen in liquid nitrogen and stored in a refrigerator at −80 °C for drought testing, two days no-watering treatment were carried out (control: water once a day). A total of 50 μmol/mL abscisic acid (ABA) (dissolved in 50 mL water) was sprayed onto plant material for 1 day as ABA treatment. Plants grown in pots were cultured at 4 °C for 1 day as cold treatment. 150 mM NaCl (dissolved in 100 mL water) was added to cultured plant pots for 1 day as salt treatment. At least three sets of biological replicates of treated *P. yunnanensis* leaves were collected for the test [66].

Total plant RNA was extracted using the RNAprep Pure Plant Plus Kit (Cat. DP441, Tiangen, Beijing, China) following the manufacturer’s instructions, using different tissues and stress treatment materials. A total of 1 μg of RNA was used for reverse transcription using the EasyScript^®^ All-in-One First-Strand cDNA Synthesis SuperMix for qPCR Reagent Kit (Transgene, Beijing, China). The relative expression levels of selected genes were measured using gene-specific primers (Table S7) and real-time quantitative PCR (qRT-PCR) analysis in a 20 μL reaction mix (TransStart^®^ Green qPCR SuperMix, Transgene, Beijing, China) with a Bio-Rad CFX96. The homolog of elongation factor 1 (EF1) in P. yunnanensis was used as the internal control [67]. The 2^−ΔΔCT^ method was used for relative expression changes of genes [42]. All qRT-PCR analyses were conducted with three replicates. The statistically significant analysis used Tukey’s LSD test (* *p* < 0.05, ** *p* < 0.01).

### 4.6. Location and Collinearity Analysis of PEAR Candidates Intraspecific and Interspecies

The chromosomal locations of the 43 candidate PEAR genes were mapped using Gene location visualize package in TBtools (v1.098691) [64]. The collinearity analysis of candidate PEAR genes among P. yunnanensis (intraspecific) and between P. yunnanensis and A.thaliana (interspecies) were obtained using MCScanX Wrapper packages in TBtools (v1.098691) [64].

### 4.7. Prediction of the Interaction Proteins and Collinearity Analysis of PEAR Candidates in P. yunnanensis

The potential interaction proteins of *P. yunnanensis* PEAR candidate proteins were predicted using the STRING server (version 12.0, https://string-db.org; accessed on 7 April 2023) [68]. Protein–protein interactions (PPIs) and functional annotation were analyzed using *Populus alba* as a background organism according to the sequence similarity in the database. All potential interacting proteins of PEAR in *P. yunnanensis* were collected according to the predicted interaction relationships in STRING, especially those predicted from curated databases and experiment.

### 4.8. Yeast One Hybrid Assay and Yeast Two Hybrid Assays

Yeast one hybrid assay (Y1H) and yeast two hybrid assays (Y2H) were used to verify the predicted protein–gene and protein–protein interaction relationships. Y1H was performed according to the manufacturer’s protocol (Cat. No. 630491, Takara, Japan). The selected upstream promoter sequence of *Poyun15034* (cyclin) was ligated to pAbAi (Takara, Japan) and the coding sequences (CDSs) of *Poyun12572*, *Poyun00546*, and *Poyun00317* (E2F) were ligated to pGADT7 (Clontech, USA). SD/-U-L (SD/-Sec-Leu, SD dropout medium with Selenocysteine and Leucine deficiency) medium with different concentrations of Aureobasidin A (AbA, 0, 200, 400, and 800 µg/L) was used for screening. Y2H was performed according to the Matchmaker GAL4 Two-Hybrid System Libraries user manual (PT3247-1, Clontech, USA) [69]. The CDS of *Poyun39374* (E2F) was ligated to pGADT7 (AD, Clontech, USA), while the CDS of *Poyun35480* (E2F) was ligated to pGBKT7 (BD, Clontech, USA). SD/-L-T (SD-Leu-Trp, SD dropout medium with Leucine and Tryptophan deficiency), SD/-H-L-T (SD-His-Leu-Trp, SD dropout medium with Histidine, Leucine, and Tryptophan deficiency), and SD/-A-H-L-T (SD-Ade-His-Leu-Trp, SD dropout medium with Adenine, Histidine, Leucine, and Tryptophan deficiency) medium with different concentrations of Aureobasidin A (AbA, 0, 400, and 800 µg/L) were used for screening. The sequences of *P. yunnanensis* PEAR candidates used for Y1H and Y2H were amplified by PCR using Phanta Max Super-Fidelity DNA Polymerase (Vazyme, Nanjing, China). The primers used are shown in Table S7.

## 5. Conclusions

This research presented a way to identify PEAR proteins in *P. yunnanensis* using phylogeny, structure, expression, and interaction verification analyses. Through phylogenetic and structural analysis, it was found that the PEAR candidates were divided into different groups, in which motifs were conserved within the same group, such as in AtPEAR1, AtPEAR2, AtOBP2, and AtHCA2, which can be used in the initial identification of PEAR proteins. The expression patterns and qRT-PCR analyses verified the function of PEAR candidates in growth regulation in tissues where there is vigorous cell division and signal response, further verifying the authenticity and functional conservation of PEAR candidates in poplar. The predictions of and experiments on protein interactions showed that PEARs are involved in regulating plant growth through cell-cycle regulation and light signaling. Therefore, our results provide valuable information regarding the identification of PEAR proteins and their function in poplar.

## Figures and Tables

**Table 1:** Characteristics of PEAR candidates in *P. yunnanensis*.

Gene ID ^a^	Number of Amino Acids ^b^	Zf-Dof domain ^c^			Molecular Weight ^d^	PI ^e^	Location ^f^	Chromosomal Location ^g^
		Start	End	Length				
Poyun37101	312	52	110	58	34,201.15	6.43	nucl	18
Poyun31130	313	52	110	58	34,667.67	6.19	nucl	14
Poyun31438	321	32	90	58	35,219.45	7.7	nucl	14
Poyun36927	329	32	90	58	36,234.49	6.36	nucl	18
Poyun31819	255	32	90	58	28,121.26	8.78	nucl	14
Poyun02469	323	69	127	58	34,513.42	9.58	nucl	1
Poyun09605	324	29	87	58	35,482	9.25	nucl	3
Poyun19219	279	38	96	58	30,702.25	8.63	nucl	7
Poyun00813	281	38	96	58	30,940.44	8.17	nucl	1
Poyun17292	338	74	132	58	35,599.59	9.3	nucl	6
Poyun29809	344	33	91	58	37,066.87	8.28	nucl	13
Poyun13901	342	33	91	58	37,178.24	8.68	nucl	5
Poyun14043	316	52	110	58	33,785.64	9.37	nucl	5
Poyun26623	261	21	79	58	28,665.65	9.15	nucl	11
Poyun22076	345	71	129	58	36,891.26	9.22	nucl	9
Poyun09599	344	56	114	58	37,933.06	8.37	nucl	3
Poyun21042	325	30	88	58	35,740.27	9.37	nucl	8
Poyun21037	354	58	116	58	38,875.33	8.87	nucl	8
Poyun12085	300	28	86	58	24,100.95	4.77	nucl	4
Poyun13505	301	28	86	58	34,055.84	4.82	nucl	5
Poyun11000	278	36	94	58	30,617.93	9.1	nucl	4
Poyun06788	326	71	129	58	34,632.6	9.1	nucl	2
Poyun38101	327	71	129	58	34,892.83	9.32	nucl	19
Poyun18110	235	18	76	58	25,176.39	8.58	nucl	7
Poyun30052	248	33	91	58	25,498.43	8.57	mito	13
Poyun30013	248	33	91	58	25,498.43	8.57	mito	13
Poyun05571	288	10	68	58	31,891.04	6.25	nucl	2
Poyun27200	261	27	85	58	27,456.1	5.95	nucl	11
Poyun36479	397	34	92	58	32,723.99	8.19	nucl	18
Poyun09665	304	44	102	58	33,883.68	8.73	nucl	3
Poyun35699	288	10	68	58	32,217.52	5.93	nucl	17
Poyun14051	253	33	91	58	25,892.81	8.66	cyto	5
Poyun11459	253	27	85	58	25,892.81	8.66	nucl	4
Poyun21099	305	45	102	57	33,822.45	8.12	nucl	8
Poyun09476	159	40	98	58	17,696.89	9.23	nucl	3
Poyun08772	503	139	197	58	55,181.76	5.46	nucl	3
Poyun22412	500	143	201	58	54,146.23	6.29	nucl	9
Poyun16915	496	139	197	58	54,103.07	6.91	nucl	6
Poyun32726	504	147	205	58	55,096.33	5.63	nucl	15
Poyun20952	161	40	98	58	17,857.2	9.15	nucl	8
Poyun27992	493	101	159	58	53,516.07	5.5	nucl	12
Poyun24653	494	101	159	58	53,742.44	6.48	nucl	10
Poyun21040	165	43	87	44	19,267.95	10.46	nucl	8

^a^ Gene ID from *P. yunnanensis* genome [34]. ^b^ Amino acids number calculated in Expasy-ProtParam (https://web.expasy.org/protparam/, accessed on 7 April 2023). ^c^ Location of Zf-Dof domain, including length, and start and end position, was predicted with Batch CD-Search Tool (https://www.ncbi.nlm.nih.gov/Structure/bwrpsb/bwrpsb.cgi; accessed on 21 April 2023). ^d,e^ Molecular weight and isoelectric point (PI) were calculated in Expasy-ProtParam. ^f^ Subcellular localization was predicted with WOLF PSORT (https://wolfpsort.hgc.jp/; accessed on 7 April 2023). nucl, nucleus; mito, mitochondria; and cyto, cytoplasm. ^g^ Chromosomal location message from *P. yunnanensis* genome [34].

## Data Availability

Data are provided within the article and Supplementary Materials.

## Supplementary Materials

The following supporting information can be downloaded at: https://www.mdpi.com/article/10.3390/ijms241713101/s1.
