# Supplementary figures and images for: Function and Characteristic Analysis of Candidate PEAR Proteins in Populus yunnanensis
Source: Int J Mol Sci. 2023 Aug 23;24(17):13101. doi: 10.3390/ijms241713101 (PMC10488302; doi:10.3390/ijms241713101)

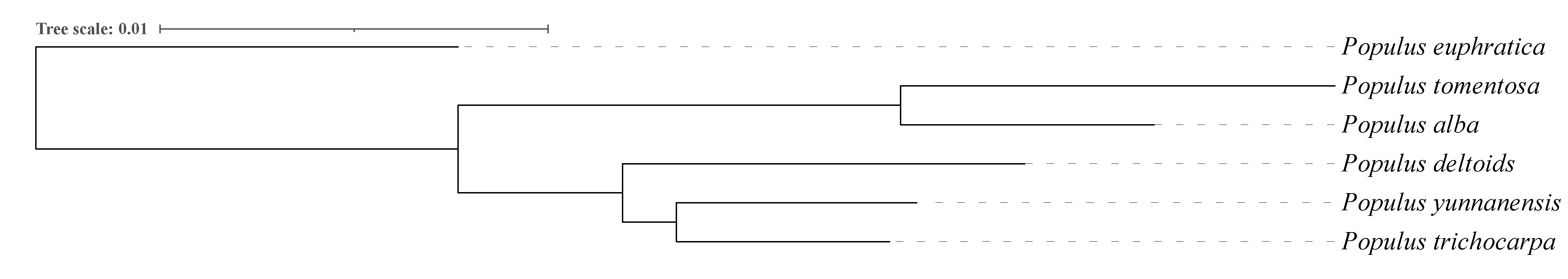

Supplement: Supplementary file 1 [file ijms-24-13101-s001.zip › Figure S1.Species tree-01.tif]
